# FSCN1 Promotes Radiation Resistance in Patients With PIK3CA Gene Alteration

**DOI:** 10.3389/fonc.2021.653005

**Published:** 2021-06-24

**Authors:** Sisi Li, Xiao-ting Huang, Meng-yao Wang, Dong-ping Chen, Ming-yi Li, Yan-yi Zhu, Yi Yu, Lu Zheng, Bin Qi, Jin-quan Liu

**Affiliations:** Department of Radiation Oncology, Affiliated Cancer Hospital & Institute of Guangzhou Medical University, Guangzhou, China

**Keywords:** fascin actin-bundling protein 1, PIK3CA, apoptosis, cervical squamous cell carcinoma, head and neck squamous cell carcinoma

## Abstract

Radiotherapy is one of the standard treatments for cervical cancer and head and neck cancer. However, the clinical efficacy of this treatment is limited by radioresistance. The discovery of effective prognostic biomarkers and the identification of new therapeutic targets have helped to overcome the problem of radioresistance. In this study, we show that in the context of PIK3CA mutation or amplification, high expression of fascin actin-bundling protein 1 (FSCN1) (using the median as the cut-off value) is associated with poor prognosis and radiotherapy response in cancer patients. Silencing FSCN1 enhances radiosensitivity and promotes apoptosis in cancer cells with PIK3CA alterations, and this process may be associated with the downregulation of YWHAZ. These results reveal that FSCN1 may be a key regulator of radioresistance and could be a potential target for improving radiotherapy efficacy in cervical cancer and head and neck cancer patients with PIK3CA alterations.

## Introduction

Cervical squamous cell carcinoma (CESC) is the second most frequent malignant tumor in females, and head and neck squamous cell carcinoma (HNSC) is the sixth most common cancer worldwide, and they are represented primarily by squamous cell carcinoma (1, 2). Almost all CESCs are associated with human papillomavirus (HPV) infection (3), while the main causes of HNSC are HPV infection, Epstein–Barr virus (EBV) infection, smoking and drinking (4). At present, the treatment of CESC and HNSC is surgery combined with adjuvant radiotherapy or radical radiation therapy (5). However, the five-year progression-free survival rate of CESC patients treated with radiotherapy and the two-year progression-free survival rate of HNSC patients treated with combined radiotherapy and cisplatin are only 43.6 (6) and 41% (7), respectively. Relapse, which causes radiotherapy failure in CESC and HNSC, is primarily attributed to radioresistance. Radioresistance is a complex process that depends on many biological factors and cellular mechanisms and is involved in proliferation, apoptosis, and the DNA damage response, which are regulated by intrinsic cell signaling networks, such as the phosphatidylinositol-3-kinase (PI3K) pathway (8). Therefore, the discovery of effective prognostic biomarkers for selecting responsive patients and the identification of new medical targets that help increase the radiosensitivity of tumors can improve the radiotherapy effects in patients.

PIK3CA is a highly frequently mutated or amplified gene in cervical cancer, head and neck cancer, breast cancer, lung cancer, neuroblastoma, and other malignant tumors (9). PIK3CA, which encodes the P110*α* catalytic subunit of PI3K, is the only tumor-specific mutated gene in the phosphatidylinositol-3 kinase (PI3K) family (10). Amplification or mutations in the PIK3CA gene that result in constitutive activation can enhance the catalytic activity of PI3K, activate the PI3K–Akt signalling pathway, and promote cell proliferation, survival, migration, and invasion. Therefore, the mutation or amplification of this gene is a driver of tumorigenesis and progression (11–13). For example, mutant PIK3CA (E542K and E545K) can enhance glucose metabolism and cell proliferation *in vitro* and *in vivo via* the *β*-catenin/SIRT3 signaling axis in cervical cancer cells (14). In addition, in head and neck squamous cell carcinoma cells, high PIK3CA gene expression was associated with activation of the Hippo–YAP pathway, which is involved in organ size, stem cell maintenance, and tumorigenesis (15).

Tumor-specific genetic alterations reveal not only the biological changes that drive tumor progression but also the vulnerabilities that can be exploited to selectively target tumors with therapeutics. Synthetic lethal therapy is a new strategy in oncotherapy that is based on the interaction between two gene deficiencies in cancer cells (16). The existence of either deficiency does not affect cell survival, but the presence of both deficiencies leads to cell death. When the first deficiency resulting from the mutation is present in tumor cells rather than in normal cells, an opportunity may exist to selectively kill cancer cells to target the second gene in tumor cells. For example, defects in the DNA mismatch repair pathway can lead to microsatellite instability (MSI) in tumor cells. Chan et al. found that deletion of the DNA helicase WRN can specifically cause double-stranded DNA fragmentation, apoptosis, and cell cycle arrest in MSI cells, which demonstrates that WRN is a potential target for synergetic death of MSI tumor cells (17). Hinze et al. used the Wnt/STOP pathway and the asparagine metabolic pathway to improve the sensitivity of leukemia to drugs. Mechanistically, by inhibiting GSK3-mediated protein ubiquitination-based degradation and maintaining continuous activation of the Wnt/STOP pathway, asparagine is reduced from metabolic sources, thus improving the effect of asparaginase (18). Although most synthetic lethality strategies are limited to basic science studies, a typical clinical application is that single-agent talazoparib (a PARP inhibitor) provides a significant benefit over standard chemotherapy for advanced breast cancer patients with a germline BRCA1/2 mutation (19). Moreover, a recent clinical study showed that the utility of the PARP inhibitor veliparib combined with carboplatin–paclitaxel improves progression-free survival in patients with germline BRCA mutation-associated advanced breast cancer (20). Therefore, the identification of genes that are synthetically lethal to PIK3CA may provide new insights into comprehensive therapy for CESC or HNSC patients with PIK3CA alterations.

In the present study, through bioinformatics analysis, we found that high FSCN1 expression indicates a poor prognosis and poor radiotherapy response in patients with PIK3CA alterations. FSCN1 is an actin-binding protein that has been reported to have pro-tumor functions. However, the synthetic lethal interaction between FSCN1 and the PI3K–Akt pathway in cancer cells remains unknown. Our study shows that FSCN1 silencing can increase the radiation sensitivity of tumor cells with PIK3CA alterations but not in cells with wild-type PIK3CA. Moreover, FSCN1 is related to apoptosis-associated genes, and its expression level is positively associated with YWHAZ specifically in tumor tissues and cells with PIK3CA alterations. This study will provide new ideas and a basis for precision treatment of cancer patients with PIK3CA alterations.

## Materials and Methods

### Acquisition of Expression and Clinicopathological Data

Gene expression profiling data, such as FPKM (fragments per kilobase per million), and clinicopathological data including radiotherapy information of patients were collected from The Cancer Genome Atlas (TCGA) cohort (https://portal.gdc.cancer.gov/). Pan-cancer gene expression profiling as TPM (transcripts per kilobase million) was obtained from the Gene Expression Profiling Interactive Analysis (GEPIA) (http://gepia.cancer-pku.cn/index.html). PIK3CA gene alteration information and overall survival rates of patients were obtained from cBioPortal (https://www.cbioportal.org/).

### Pathway Enrichment Analysis

Pearson r values were calculated for all genes expressed with FSCN1 in PIK3CA-wild-type or PIK3CA-altered patients. Gene set enrichment analysis (GSEA) was performed according to Pearson r values using Reactome gene sets. Genes with an absolute value of Pearson r greater than 0.4 were defined as FSCN1-coexpressed genes and were subsequently subjected to gene ontology (GO) enrichment analysis (Biological process) using DAVID (https://david.ncifcrf.gov/).

### Cell Culture

The HeLa and CaSki cells are cervical cancer cell lines, and MDA-MB-231 and T47D cells are breast cancer cell lines. They are all obtained from the American Type Culture Collection (ATCC, Manassas, VA). HeLa, MDA-MB-231, and T47D cells were maintained in Dulbecco’s Modified Eagle’s Medium (DMEM) containing 10% fetal bovine serum (FBS; Gibco, Melbourne, Australia). CaSki cells were cultured in Roswell Park Memorial Institute (RPMI)-1640 medium containing 10% FBS. All cells were maintained in a humidified atmosphere of 5% CO_2_ at 37°C.

### siRNA Transfection

FSCN1 siRNAs (si-1: 5′-GGUCAACAUCUACAGCGUCACdTdT; si-2: 5′-GCGCCUACAACAUCAAAGACUdTdT) and YWHAZ siRNAs (si-1: 5′-GCTCGAGAATACAGAGAGAdTdT; si-2: 5′-CGCTGGTGATGACAAGAAAdTdT) were purchased from GenePharma (Guangzhou, China). Cells were seeded in 24-well culture plates and transfected with siRNAs using Lipofectamine RNAiMAX reagent (Invitrogen) according to the manufacturer’s protocol. After 24 h of transfection, the cells were used for functional assays.

### Radiation Colony Formation Assay

Cells were trypsinized to generate single-cell suspensions and seeded in six-well culture plates (pretreated with siRNAs for 24 h) in triplicate. After 24 h, cells were exposed to the indicated doses of irradiation using 6-MV X-rays from linear accelerators (Varian2300EX; Varian, Palo Alto, CA, USA) at a dose rate of 5 Gy/min. Ten to fourteen days after radiation, colonies were fixed in methanol/acetic acid and stained with 0.5% crystal violet. Colonies were counted using the naked eye, and the surviving fraction (SF) was calculated.

### Analysis of Gene Expression

The expression level of target genes was analyzed by real-time quantitative polymerase chain reaction (qPCR) or western blotting. Total RNA was extracted using TRIzol (Life Technologies, Carlsbad, CA, USA). qRT-PCR was performed to detect the expression levels of FSCN1 (F: CCAGGGTATGGACCTGTCTG; R: GTGTGGGTACGGAAGGCAC) and YWHAZ (F: TGTAGGAGCCCGTAGGTCATC; R: GTGAAGCATTGGGGATCAAGA) using the SYBR Green PCR kit from Takara Biotechnology (Takara, Dalian, China). GAPDH was used as an endogenous control. Western blotting was used to examine protein levels in cell lines. Cellular proteins were separated on SDS-polyacrylamide gels, electrophoretically transferred to polyvinylidene difluoride membranes, and then detected with the indicated antibodies. The antibodies were a rabbit polyclonal antibody (pAbs) against caspase-3 (1:1,000; A2156; ABclonal Biotechnology Co., Ltd., Wuhan, China) and a rabbit mAb against *β*-actin (1:5,000; 8457S; Cell Signaling Technology, Beverly, MA, USA).

### Statistical Analysis

The difference in FSCN1 expression between each group was analyzed using Student’s t test. Recurrence-free survival (RFS) was calculated from the date of tumor resection to the time of first recurrence. For survival analysis of 2,845 candidate genes (including FSCN1) in PIK3CA-wild-type or PIK3CA-altered patients, log-rank tests between the high and low expression groups (using the medians as cut-off values for each gene) were performed using the survival package in R software, and a *P* value of less than 0.05 was considered statistically significant. Kaplan–Meier survival curves were plotted using GraphPad Prism version 5.0 software (GraphPad Software, Inc., San Diego, CA, USA). Cox proportional hazard regression analyses were performed using SPSS version 16.0 software (IBM SPSS, Inc., Chicago, IL, USA).

## Results

### FSCN1 Expression Is Associated With Prognosis and Response to Radiotherapy in PIK3CA-Altered Patients

**Figure 1:**
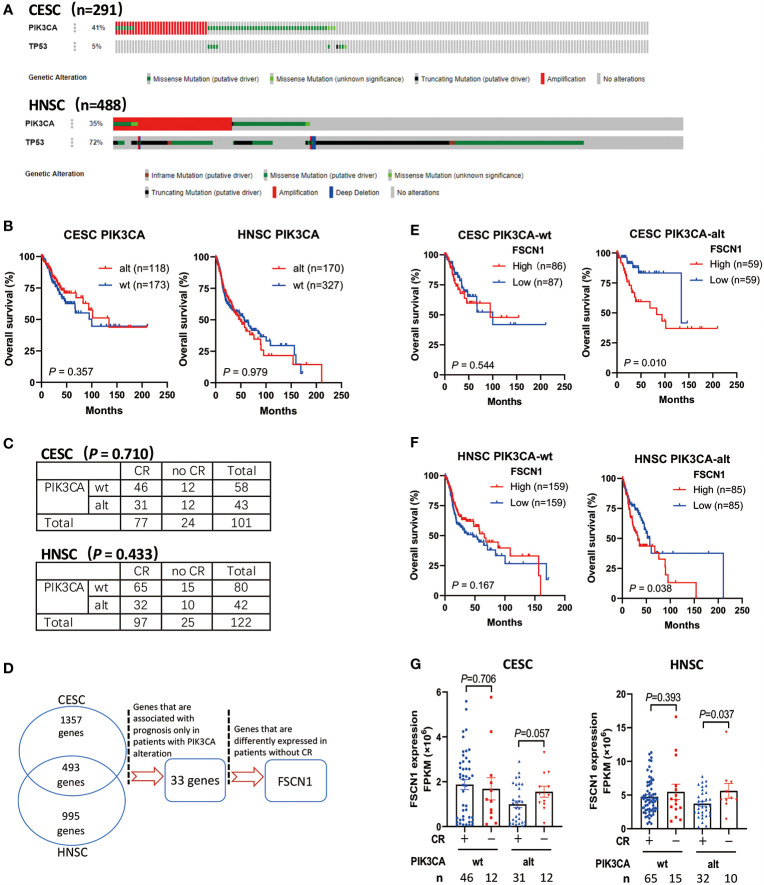
High expression of FSCN1 is associated with poor prognosis and response to radiotherapy in CESC and HNSC patients with PIK3CA alterations. **(A)** The frequency of PIK3CA gene alterations in cervical cancer (CESC) and head and neck cancer (HNSC) cases from the cBioPortal database. **(B)** Kaplan–Meier curve shows the overall survival (OS) rate in CESC and HNSC cases from the TCGA cohort with wild-type PIK3CA or altered PIK3CA. The *P* values were calculated by log-rank test. **(C)** The table shows the radiotherapy response in CESC and HNSC with wild-type PIK3CA or altered PIK3CA. **(D)** The workflow of screening genes that have synthetic lethal interactions with PIK3CA using TCGA Data Portal. **(E, F)** Kaplan–Meier curve shows that the overall survival (OS) rate was significantly lower in the high FSCN1 group with PIK3CA activation in the TCGA cohort. The median FSCN1 level was chosen as the cut‐off value for separating the FSCN1 high‐level and low-level groups. **(G)** The expression of FSCN1 in different CESC and HNSC patient groups with or without a complete response to radiotherapy. PIK3CA “wt” or “alt”, PIK3CA wild type or altered (mutated or amplificated); “CR” or “no CR”, complete response or no complete response. Data are presented as means ± SEM.

Through the analysis of TCGA data of patients with cervical cancer (291 cases) and head and neck cancer (488 cases), we found that PIK3CA was a highly altered gene (including activating mutations and amplification). The proportion of cervical cancer and head and neck cancer patients with mutations and amplifications accounted for 41 and 35% of cases, respectively (**Figure 1A**). However, PIK3CA alterations were not associated with the overall survival of cervical cancer patients (*P* = 0.357) or head and neck cancer patients (*P* = 0.979) (**Figure 1B**), and it was also not associated with complete response to radiotherapy (**Figure 1C**). According to the synthetic lethal theory of tumor cells, alterations in the PIK3CA oncogene may lead to potential defects in tumor cells so that they would be specifically dependent on the activation of certain pathways or on the expression of certain genes. Therefore, based on whether PIK3CA is altered, do other genetic alterations indicate changes in prognosis and response to radiotherapy and chemotherapy?

To screen the candidate genes that have synthetic lethal interactions with the PIK3CA gene (**Figure 1D**), we first analyzed TCGA expression data through the GEPIA database (http://gepia.cancer-pku.cn/) and selected genes whose expression in cervical tumors or head and neck tumors was up-regulated two-fold compared with normal tissues (union set). In all, 2,845 candidate genes were analyzed. Next, we analyzed the correlation between the expression levels of these genes and the prognosis of patients with different PIK3CA statuses and screened genes that can specifically indicate the prognosis of the PIK3CA-altered group (33 genes, **Supplementary Table S1**). Finally, we analyzed the expression of genes in different radiotherapy response groups, and we further selected genes whose expression was up-regulated in patients who did not exhibit a complete response to radiotherapy. We found that high expression of the FSCN1 gene in patients with PIK3CA alterations was associated with worse overall survival (cervical cancer, *P* = 0.010, **Figure 1E**; head and neck cancer, *P* = 0.038, **Figure 1F**). However, in patients without PIK3CA alterations, the expression level of FSCN1 was not related to patient prognosis (**Figures 1E, F** **)**. Also, both univariate and multivariate analyses confirmed FSCN1 up-regulation as an independent risk factor for poorer survival in patients with PIK3CA alterations but not in patients with wild-type PIK3CA (**Table 1**). Furthermore, in cancer patients with PIK3CA alterations, FSCN1 was downregulated in patients who had a complete response to radiotherapy (cervical cancer, *P* = 0.057; head and neck cancer, *P* = 0.037, **Figure 1G**). This suggests that FSCN1 may play a key role in PIK3CA-altered tumor cells and that the survival of PIK3CA-altered tumor cells under radiotherapy treatment may be specifically accompanied by high expression of the FSCN1 gene.

**Table 1:** Univariate and multivariate analysis of factors associated with overall survival.

A. CE SC PIK3CA-wt			
Clinical Variables	Case Number	HR (95%CI)	*P*
**Univariate analysis**			
Age (>=50 years vs. (<50 years)	62/111	1.172 (0.626-2.196)	0.62
TNM (II/III/IV vs. 0/I)	83/65	2.040 (0.963-4.324)	0.063
Stage (II/III/IV vs. I)	76/95	1.134 (0.604-20129)	0.696
Grade (GI/2 vs. G3/x)	77/90	1.191 (0.631-2.245)	0.589
FSCNl (High vs. Low)	86/87	1.212 (0.651-2.258)	0.545
**Multivariate analysis**			
TNM (II/III/IV vs. 0/I)	83/65	2.206 (1.016-4. 791)	**0.045**
**B. CESC PIK3CA-alt**			
**Clinical Variables**	**Case Number**	**HR (95%CI)**	***p***
**Univariate analysis**			
Age (>=50 years vs. (<50 years)	59/59	1.233 (0.575-2.641)	0.59
TNM (II/III/IV vs. 0/I)	61/38	2.505 (0.834-7.520)	0.102
Stage (II/III/IV vs. I)	54/60	2.018 (0.933-4.365)	0.075
Grade (GI/2 vs. G3/x)	69/47	0.630 (0.288-1.378)	0.247
FSCNl (High vs. Low)	59/59	2.981 (1.248-7.122)	**0.014**
**Multivariate analysis**			
FSCNl (High vs. Low)	59/59	3.646(1.298-10.241)	**0.014**
**C. HNSC PIK3CA-wt**			
**Clinical Variables**	**Case Number**	**HR (95%CI)**	***p***
**Univariate analysis**			
Age (>=50 years vs. (<50 years)	268/50	0.897 (0.561-1.433)	0.649
Gender (male vs. female)	231/87	0.837 (0.580-1.208)	0.343
Stage (IV vs. I/II/III)	183/135	1.695 (1.187-2.422)	**0.004**
Grade (GI/2 vs. G3/4/x)	225/91	0.994 (0.685-1.444)	0.976
FSCNl (High vs. Low)	159/159	1.272 (0.903-1.792)	0.168
**Multivariate analysis**			
Stage (IV vs. I/II/III)	231/87	1.687 (1.180-2.412)	**0.004**
**D. HNSC PIK3CA-alt**			
**Clinical Variables**	**Case Number**	**HR (95%CI)**	***p***
**Univariate analysis**			
Age (>=50 years vs. (<50 years)	147/23	1.122 (0.575-2. 192)	0.735
Gender (male vs. female)	128/42	0.604 (0.372-0.981)	**0.042**
Stage (IV vs. I/II/III)	112/58	1.423 (0.857-2.361)	0.172
Grade (G1/2 vs. G3/4/x)	126/43	1.344 (0.788-2.291)	0.277
FSCNl (High vs. Low)	85/85	1.620 (1.022-2.567)	**0.04**
**Multivariate analysis**			
FSCNl (High vs. Low)	85/85	1.611 (1.016-2.553)	**0.042**

HR and P values were ca lcula ted by Cox proportional hazards regression using TCGA data. Factors were included in the model according to “Forward LR” algorithm. CI, confidence interval; HR, hazard ratio;. PIK3CA-wt, wild type PIK3CA; PIK3CA-alt, mutated or amplificated PIK3CA. The P values less than 0.05 were shown in bold.

### Silencing FSCN1 Enhances Sensitivity to IR Treatment and Promotes Apoptosis in Cancer Cells With PIK3CA Alterations

**Figure 2:**
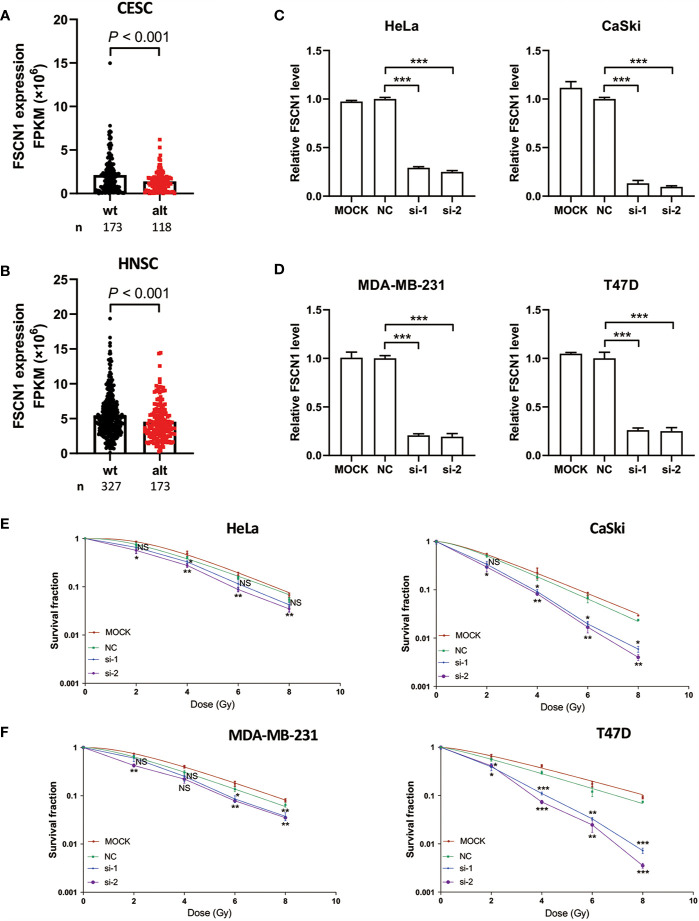
FSCN1 silencing enhances sensitivity of cancer cells with PIK3CA alterations to IR treatment. **(A, B)** The expression of FSCN1 in PIK3CA-altered and PIK3CA-wild-type patients from the GEPIA database. **(C, D)** The knockdown efficiency of siFSCN1s. Relative expression of FSCN1 in HeLa, CaSki, MDA-MB-231, and T47D cells was detected using qRT-PCR. **(E, F)** Knockdown of FSCN1 sensitizes cancer cells to IR treatment. Colony formation assays of HeLa, CaSki, MDA-MB-231, and T47D cells transfected with FSCN1 siRNA. Surviving fractions were determined based on plating efficiencies. Plating efficiency of Hela, Caski, T47D, MDA-MB-231 is 62.3, 54.16, 63, and 46.16%. Data are presented as the means ± SEM of at least three independent experiments. *P < 0.05; **P < 0.01; ***P < 0.001. NS, not significant.

To explore the function of FSCN1 in tumor biology, we first analyzed the characteristics of FSCN1 gene expression. We found that compared with cancer-adjacent normal tissues, FSCN1 expression was up-regulated in a variety of tumor tissues, such as gastric cancer, breast cancer, lung cancer, and kidney cancer, in the TCGA database. This suggests that FSCN1 may function as an oncogene (**Supplementary Figure S1**). In addition, we found that FSCN1 gene expression was lower in patients with cervical cancer (*P* < 0.001) (**Figure 2A**) and head and neck tumors (*P* < 0.001) (**Figure 2B**) with PIK3CA alterations than in those in the wild-type group. This suggests that PIK3CA may regulate FSCN1 expression.

To examine the mechanism of FSCN1 in cancer cells under IR treatment, we performed colony formation assays in a panel of cancer cell lines, including HeLa (a cervical adenocarcinoma-derived PIK3CA-WT cell line), CaSki cells (a cervical squamous cell carcinoma cell line that is heterozygous for PIK3CA-E545K) (**Figure 2C**), MDA-MB-231 (a breast cancer PIK3CA-WT cell line) and T47D (a breast cancer cell line that harbors the H1047R mutation with amplified PIK3CA) (**Figure 2D**). The results showed that FSCN1 knockdown significantly decreased the growth of these cancer cells under IR treatment except for si-1 transfection in HeLa cells. Intriguingly, compared with PIK3CA-WT cell lines (HeLa and MDA-MB-231), a relatively lower surviving fraction of cells with PIK3CA alterations (CaSki and T47D) was observed when FSCN1 was silenced in response to IR (**Figures 2E, F** **)**.

**Figure 3:**
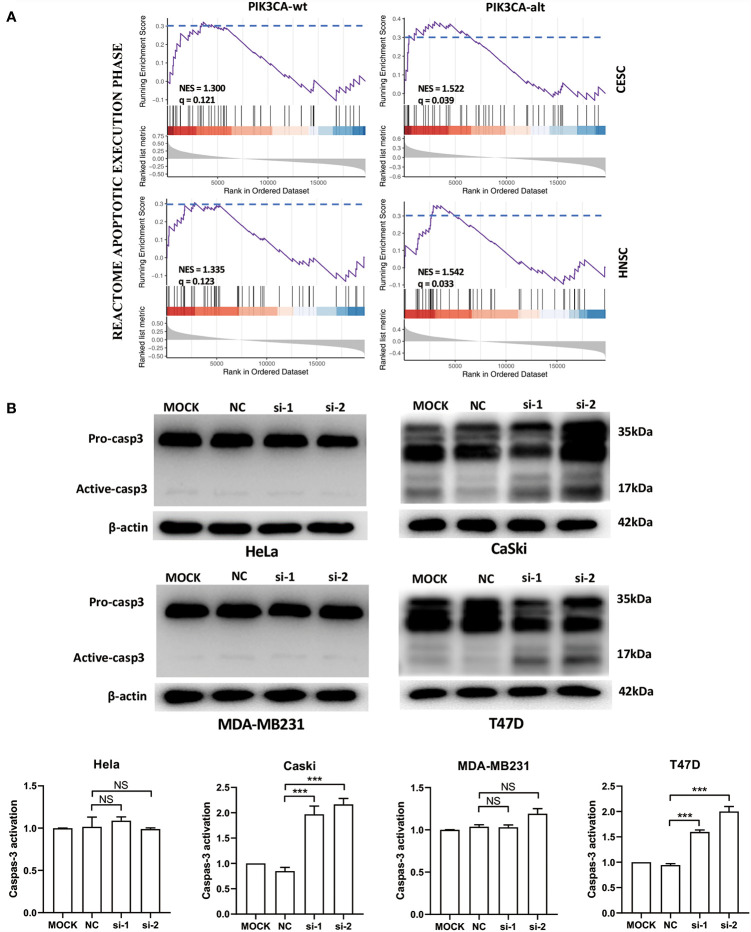
FSCN1 inhibits apoptosis of cancer cells with PIK3CA alterations. **(A)** FSCN1-correlated genes were enriched in apoptosis-related pathways in patients with PIK3CA alterations. The plots show the results of GSEA of the apoptosis pathway of the Reactome database. NES, normalized enrichment score; q, false discovery rate; PIK3CA-wt, wild-type PIK3CA; PIK3CA-alt, mutated or amplified PIK3CA. **(B)** Western blotting analysis of caspase-3 expression in HeLa, CaSki, MDA-MB-231 and T47D cells transduced with FSCN1 siRNA, after 4 Gy IR treatment. Quantified levels of active-caspase-3 are shown. Data are presented as means ± SEM. ***P < 0.001. NS, not significant.

To analyze the function of the FSCN1 gene in tumors with different types of PIK3CA alterations, we calculated the correlation between the expression level of FSCN1 and that of other genes in PIK3CA-wild-type or PIK3CA-altered patients. GSEA analysis according to correlation coefficient showed that FSCN1-correlated genes in patients with PIK3CA alteration got a larger enrichment score with statistical significance than that in PIK3CA wild type patients (NES: 1.52 and 1.54 *vs* 1.30 and 1.33; FDR: 0.04 and 0.03 *vs* 0.12 and 0.12; set FDR<0.05 as statistically significant) (**Figure 3A**), suggesting that the function of FSCN1 were relevant to apoptosis pathway in patients with PIK3CA alteration, but not in PIK3CA wild type patients. This suggested that PIK3CA-altered tumor cells may specifically rely on FSCN1 to survive radiotherapy treatment. Consistently, the detection of cleaved caspase-3 indicated that FSCN1 knockdown promoted IR-induced apoptosis in CaSki and T47D cells but not in HeLa and MDA-MB-231 cells (**Figure 3B**).

### FSCN1 May Regulate the Expression of YWHAZ in the Context of PIK3CA Alterations

**Figure 4:**
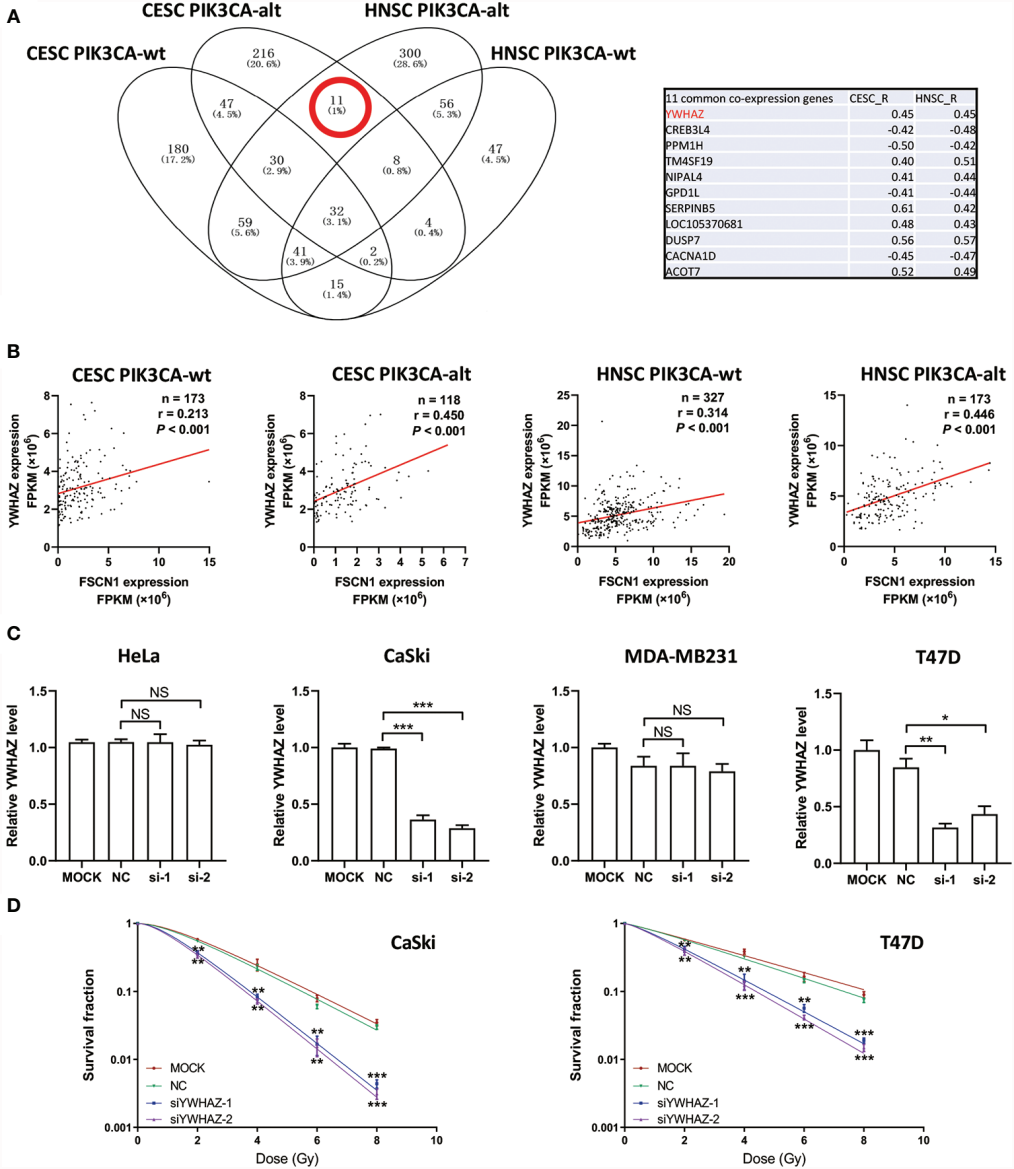
FSCN1 regulates the expression of YWHAZ in cancer cells with PIK3CA alterations. **(A)** Venn diagram shows FSCN1-correlated genes in CESC and HNSC patients with wild-type or altered PIK3CA. Eleven genes were coexpressed with FSCN1 in the PIK3CA-altered group of cervical cancer and head and neck cancer patients. Pearson correlation coefficient values are shown in the table. **(B)** Pearson correlation analysis for the expression levels of FSCN1 and YWHAZ. Graphs show a stronger positive correlation between FSCN1 and YWHAZ in patients with altered PIK3CA than in those with wild-type PIK3CA according to TCGA data. **(C)** Relative expression of YWHAZ analyzed by qRT-PCR in HeLa, CaSki, MDA-MB-231, and T47D cells transduced with FSCN1 siRNAs. **(D)** Knockdown of YWHAZ sensitizes cancer cells to IR treatment. Colony formation assays of CaSki and T47D cells transfected with YWHAZ siRNA. Surviving fractions were determined based on plating efficiencies. PIK3CA-wt, wild type PIK3CA; PIK3CA-alt, mutated or amplificated PIK3CA. For **(C, D)**, data are presented as the means ± SEM of at least three independent experiments. *P < 0.05; **P < 0.01; ***P < 0.001. NS, not significant.

**Figure 5:**
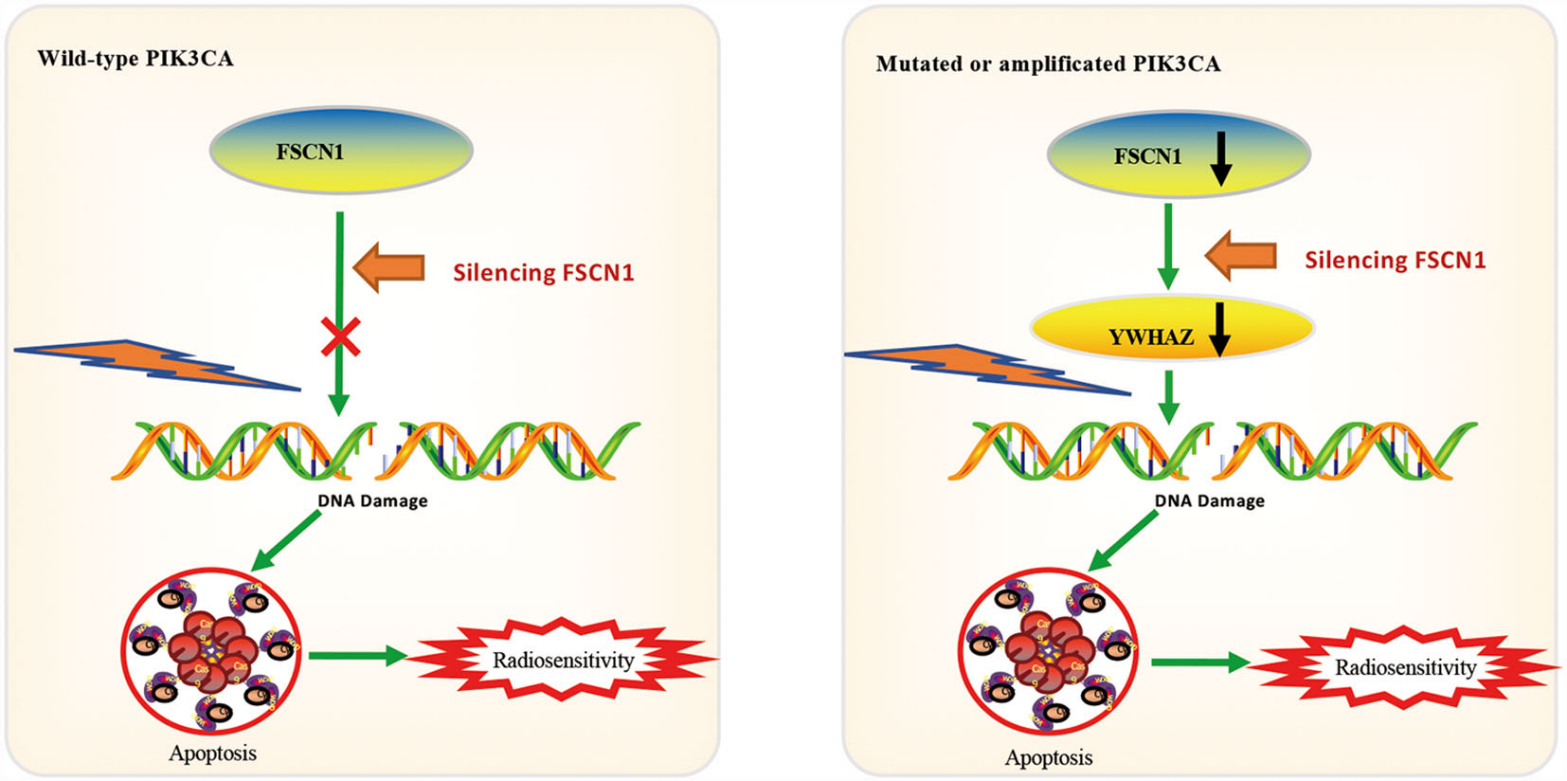
Model of mechanisms of FSCN1 in PIK3CA-altered tumor cells.

To explore the potential downstream pathways of FSCN1 by selecting genes correlated only with FSCN1 expression in either PIK3CA-wild-type or PIK3CA-altered patients (|r| >0.4) for the GO analysis (**Supplementary Figure S2A**), we found that the same signaling pathway (“protein targeting”) was enriched according to the gene function enrichment analysis in PIK3CA-altered patients in both cancer types (**Supplementary Figure S2B**). However, in PIK3CA-wild-type patients, no consistent signaling pathway was observed between the two types of tumors when gene function enrichment analysis was performed. Interestingly, we found 11 genes that were correlated with FSCN1 only in the PIK3CA-altered group in both cervical cancer and head and neck cancer (**Figure 4A**). Among them, YWHAZ (coding the protein 14-3-3*ζ*) was attributed to the “protein targeting” gene set of GO. The correlation between the expression levels of FSCN1 and YWHAZ was significantly stronger in patients with PIK3CA alterations than in patients with wild-type PIK3CA (r: 0. 450 > 0.213, p = 0.013; r: 0.446 > 0.314, p = 0.051) (**Figure 4B**). Furthermore, FSCN1 knockdown significantly downregulated the expression of YWHAZ in CaSki and T47D cells but not in the PIK3CA-WT HeLa and MDA-MB-231 cells (**Figure 4C**). Consistent with silencing FSCN1, YWHAZ knockdown also decreased the surviving fraction of CaSki and T47D cells (with altered PI3KCA) in response to IR (**Figure 4D**). These results suggested that in cervical tumors and head and neck tumors with PIK3CA alterations, the pro-tumor function of FSCN1 may be mediated by 14-3-3*ζ*, which modulates signal transduction by binding to phosphoserine-containing proteins (**Figure 5**).

## Discussion

Since radiotherapy resistance is still the main cause of radiotherapy failure in cervical cancer and head and neck cancer, the discovery and verification of novel biomarkers that indicate the response to radiotherapy are urgently needed for individualized treatment. By determining the relationship between specific gene expression and the mutation status of related genes, the radiosensitivity of patients can be predicted earlier and more accurately, which can improve patient prognosis. We analyzed TCGA clinical data and found high-frequency mutations or amplifications of the PIK3CA gene in patients with cervical cancer and head and neck cancer. However, pure PIK3CA gene alterations are not correlated with the overall survival of patients or the response to radiotherapy. Next, we found that higher expression of the FSCN1 gene in cervical cancer and head and neck cancer patients with PIK3CA alterations was associated with poorer overall survival and radiotherapy response. Then, we found that expression of the FSCN1 gene was up-regulated in a variety of cancers compared with adjacent tissues, which suggested that the FSCN1 gene may be an oncogene. *In vitro* experiments showed that FSCN1 silencing sensitized tumor cells with PIK3CA alterations, but not cells with wild-type PIK3CA to IR. Furthermore, using bioinformatics analysis in cervical cancer and head and neck cancer patients with PIK3CA alterations, FSCN1-co-expressed genes were enriched in the apoptosis pathway. Consistently, FSCN1 knockdown promoted cell apoptosis in tumor cells with PIK3CA alterations. This leads us to propose a hypothesis that FSCN1 may have a pro-tumor function among patients with PIK3CA alterations. FSCN1 may be a synthetic lethality gene with PIK3CA and its expression level may serve as a biomarker for prognosis and radiotherapy response in cervical cancer and head and neck cancer.

Our study found that the FSCN1 gene is related to radiosensitivity and is associated with therapeutic efficacy and prognosis in cervical cancer and head and neck cancer patients with PIK3CA alterations. In recent decades, FSCN1 has been demonstrated to be an effective marker of poor prognosis and a potential therapeutic target in many invasive tumors (21–23). FSCN1 codes for important structures within actin cytoskeletal protrusions, which can promote the organization of two main actin-based structures: cortical cell protrusions that mediate cell interaction and migration and cytoplasmic microfilaments that contribute to cell structure and intracellular movement (24, 25). Studies have shown that FSCN1 is often located at the invasion boundary of tumor cell nests. From diffuse expression to concentrated expression, FSCN1 changes the morphology of cell membrane protrusions, thereby reducing intercellular adhesion, which enables the subgroups located at the tumor boundary to invade the surrounding tissue; this in turn increases tumor cell migration and invasion (26, 27). FSCN1 expression in breast cancer has also been shown to be a key feature that supports transendothelial migration, which is a key step in the metastasis process (28). In addition, FSCN1 can act as a mediator of self‐seeding of metastatic circulating tumor cells (CTCs); FSCN1 can also mediate the invasiveness of tumor cells *in vitro* and metastasis *in vivo*, regroup the primary tumor, and assist in the completion of “self-seeding” (29). FSCN1 regulates the expression of some genes related to invasion, such as N-cadherin, E-cadherin and MMP9. However, the exact mechanism of FSCN1 signalling is still unknown and requires further study. Mechanistically, FSCN1 expression leads to the up-regulation of MMP9 and N-cadherin. Therefore, FSCN1 may be a biomarker in cervical cancer and head and neck cancer patients at a high risk of metastasis.

Our study also reveals that FSCN1 expression is correlated with the expression of YWHAZ, which encodes the 14-3-3*ζ* protein, and that silencing FSCN1 downregulates YWHAZ only in tumor cells with PIK3CA alterations. This suggests that FSCN1 may have a new function in regulating 14-3-3*ζ* in the background of PI3K–Akt pathway activation. 14‐3‐3*ζ* belongs to the 14-3-3 protein family and can form homo/heterodimers that bind with phosphorylated serine/threonine motifs on their target proteins, thereby altering the activity of their targets through post‐translational regulation. It has been widely reported that 14‐3‐3*ζ* acts as an oncogene by promoting proliferation, survival and migration/invasiveness of tumor cells (30–32). Moreover, 14‐3‐3*ζ* can modulate PI3K/Akt pathway activity. For example, 14-3-3*ζ* can induce hyperactivation of the PI3K/Akt pathway, result in increased p53 degradation and confer breast cancer cells with resistance to anoikis (33). 14-3-3*ζ* also controls Akt Thr308 phosphorylation in an intestinal inflammation model (34). Interestingly, IR-induced 14-3-3*ζ* promotes invasion of ErbB2-positive breast cancer cells, which display enhanced downstream PI3K/Akt signaling activity (35); this aligns with our hypothesis that 14-3-3*ζ* and its potential regulator FSCN1 will be ideal therapeutic targets in PI3K/Akt-overactive tumor cells.

In our results, the multivariate Cox analysis showed that the expression of FSCN1 can act as an independent prognostic factor in PIK3CA altered patients. To confirm the potential of FSCN1 as a predictor for prognosis in cancer patients, we also performed ROC analysis (**Supplementary Figure S3**). The results showed that the FSCN1 expression had a larger AUC in PIK3CA-altered patients than in PIK3CA wild-type patients. Although, AUC is still not significant enough to support FSCN1 as a single factor to predict prognosis in PIK3CA-altered patients, this result can give evidence of the different functions of FSCN1 in different contexts of PIK3CA genotype. In fact, in our screening pipeline, we only considered the candidate genes which was up-regulated in tumor tissues and identified one gene (FSCN1) that was associated with prognosis and response to radiotherapy in PIK3CA-altered patients. Actually, the genes that are not up-regulated in tumor tissues might also perform similarly to FSCN1, so in the future we can identify more other genes that are associated with prognosis and response to radiotherapy in PIK3CA-altered patients and build up a practical predictor model with more accuracy. Moreover, further *in vitro* and *in vivo* experiments are required to determine the mechanism by which FSCN1 regulates YWHAZ expression and cell apoptosis.

In summary, we show that the high expression of FSCN1 indicates poor prognosis and radiotherapy response of cancer patients with PIK3CA alterations. Silencing FSCN1 promotes apoptosis of cancer cells with PIK3CA alterations, thus enhancing radiosensitivity, and this process may be associated with the downregulation of YWHAZ.

## Data Availability Statement

The original contributions presented in the study are included in the article/**Supplementary Material**. Further inquiries can be directed to the corresponding authors.

## Author Contributions

SL designed this study, collected data, and performed the analysis. X-tH performed the experiments. SL and M-yW wrote the manuscript. BQ, D-pC, M-yL, Y-yZ, YY, and LZ reviewed and corrected the manuscript. J-qL supervised this project and provided the fund. All authors contributed to the article and approved the submitted version.

## Conflict of Interest

The authors declare that the research was conducted in the absence of any commercial or financial relationships that could be construed as a potential conflict of interest.

## Supplementary Material

Supplementary Figure 1 FSCN1 expression profiling in pan-cancer from the GEPIA database.

Supplementary Figure 2 FSCN1-correlated genes are enriched in the GO pathway “protein targeting” only in patients with altered PIK3CA. **(A)** Venn diagram shows FSCN1-correlated genes in CESC and HNSC patients. **(B)** Gene ontology (GO) enrichment analysis of FSCN1-coexpressed genes in PIK3CA-wild-type or PIK3CA-altered patients using DAVID. PIK3CA-wt, wild type PIK3CA; PIK3CA-alt, mutated or amplificated PIK3CA.

Supplementary Figure 3 Performance of FSCN1 level to predict prognosis in PIK3CA wild-type or altered patients. ROC for performance at 12, 36, 60, and 84 months in cervical cancer **(A, B)** and head and neck cancer **(C, D)** patients. TP, true positive value; FP, false positive value; wt, wild-type PIK3CA; alt, mutated or amplificated PIK3CA.
