# Supplementary figures and images for: FSCN1 Promotes Radiation Resistance in Patients With PIK3CA Gene Alteration
Source: Front Oncol. 2021 Jun 24;11:653005. doi: 10.3389/fonc.2021.653005 (PMC8264437; doi:10.3389/fonc.2021.653005)

Supplementary figure S1

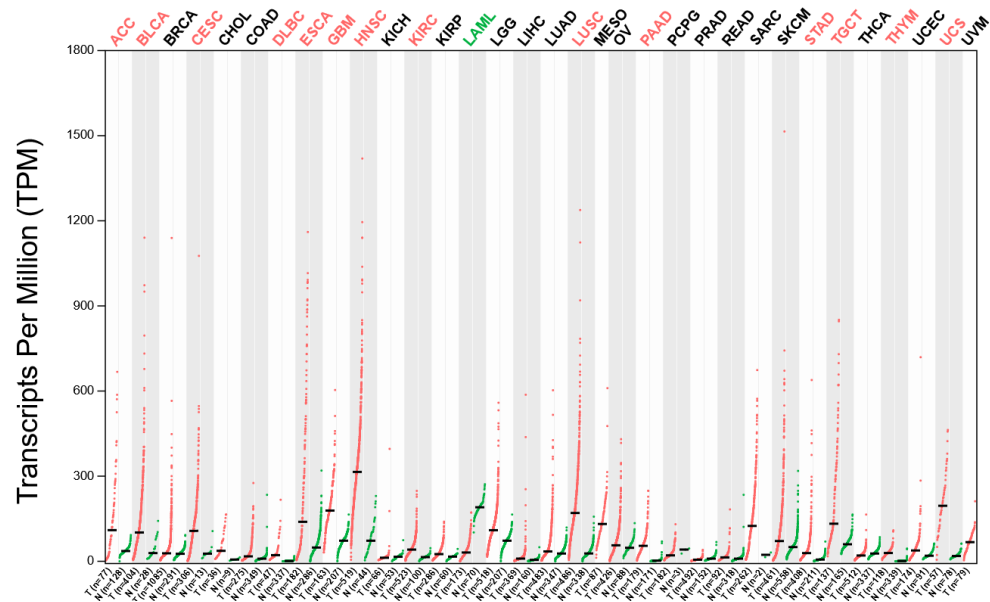

Supplement: Supplementary Figure 1 — FSCN1 expression profiling in pan-cancer from the GEPIA database. [file Image_1.pdf]

Supplementary figure S2

A

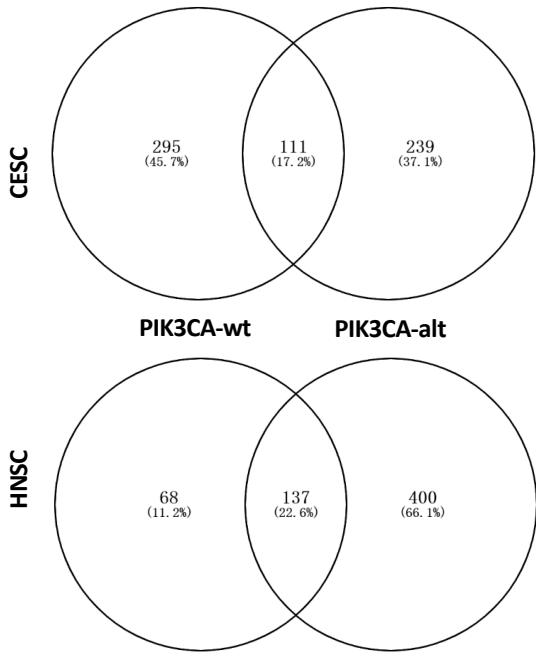

B

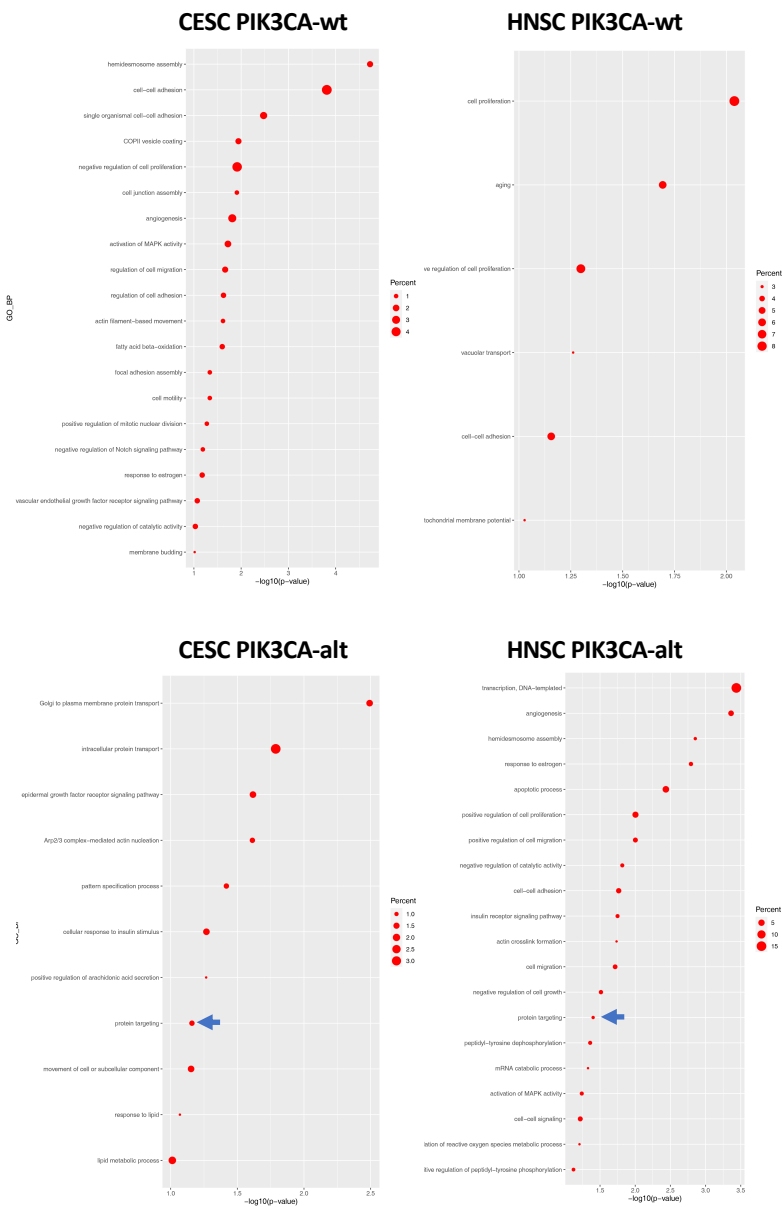

Supplement: Supplementary Figure 2 — FSCN1-correlated genes are enriched in the GO pathway “protein targeting” only in patients with altered PIK3CA. (A) Venn diagram shows FSCN1-correlated genes in CESC and HNSC patients. (B) Gene ontology (GO) enrichment analysis of FSCN1-coexpressed genes in PIK3CA-wild-type or PIK3CA-altered patients using DAVID. PIK3CA-wt, wild type PIK3CA; PIK3CA-alt, mutated or amplificated PIK3CA. [file Image_2.pdf]

Supplementary figure S3

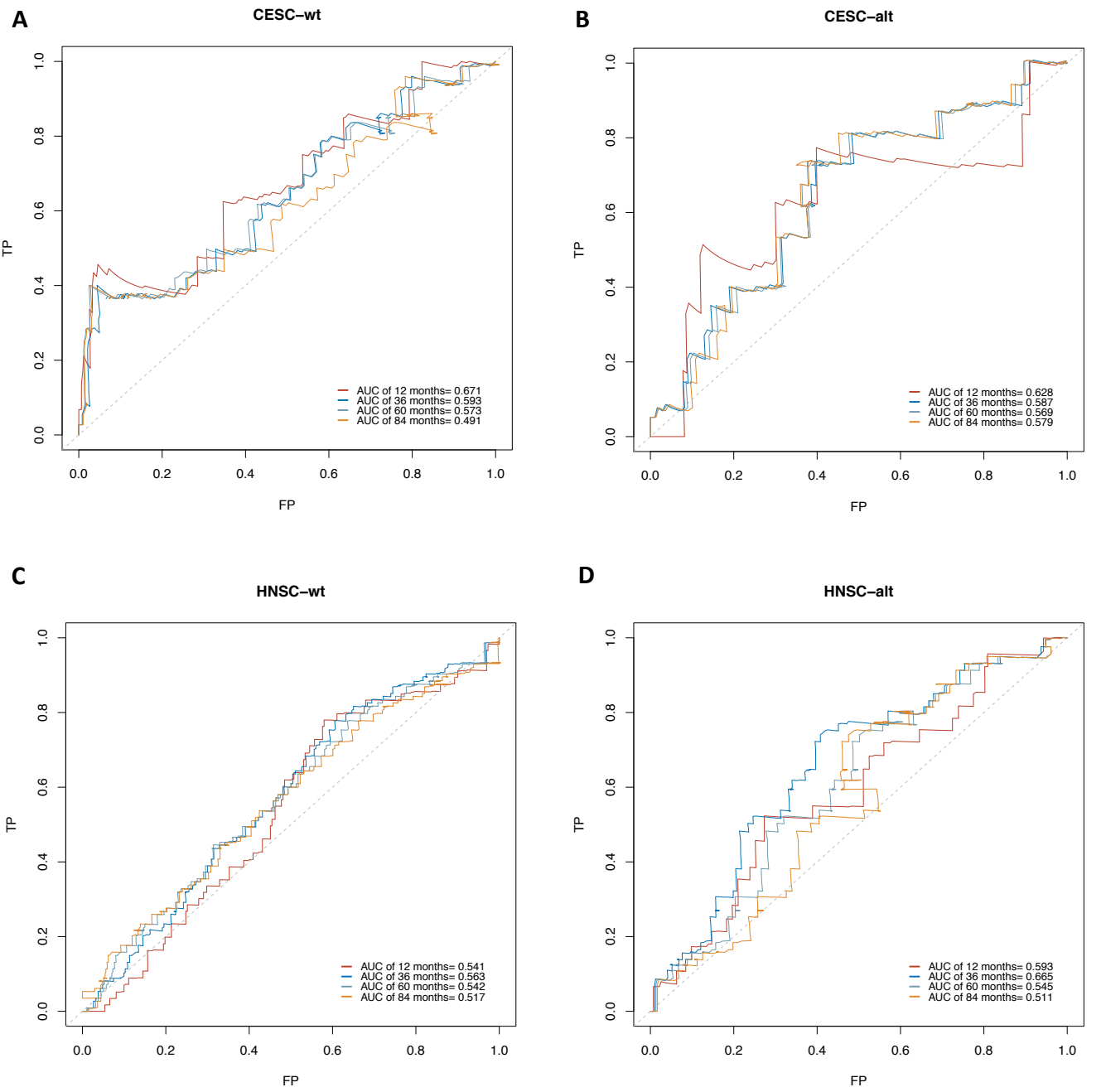

Supplement: Supplementary Figure 3 — Performance of FSCN1 level to predict prognosis in PIK3CA wild-type or altered patients. ROC for performance at 12, 36, 60, and 84 months in cervical cancer (A, B) and head and neck cancer (C, D) patients. TP, true positive value; FP, false positive value; wt, wild-type PIK3CA; alt, mutated or amplificated PIK3CA. [file Image_3.pdf]
