# Supplementary material for: FSCN1 Promotes Radiation Resistance in Patients With PIK3CA Gene Alteration
Source: Front Oncol. 2021 Jun 24;11:653005. doi: 10.3389/fonc.2021.653005 (PMC8264437; doi:10.3389/fonc.2021.653005)
Supplement: Supplementary file 4 [file Table_1.docx]

**Supplementary table 1. The expression level of genes that are associated with prognosis in PIK3CA altered patients**

| Gene Symbol | CESC | | | | HNSC | | | |
| --- | --- | --- | --- | --- | --- | --- | --- | --- |
|  | PIK3CA_wt | | PIK3CA_alt | | PIK3CA_wt | | PIK3CA_alt | |
|  | CR | no CR | CR | no CR | CR | no CR | CR | no CR |
| **FSCN1** | **18.7^*^** | **16.8** | **9.9** | **15.5** | **47.3** | **54.7** | **37.2** | **56.2** |
| ITGA5 | 3.5 | 5.5 | 1.8 | 2.6 | 6.8 | 7.1 | 5.3 | 10.0 |
| FKBP14 | 0.8 | 0.7 | 0.8 | 0.9 | 1.2 | 1.0 | 1.0 | 1.8 |
| PMEPA1 | 5.8 | 9.0 | 4.4 | 6.2 | 8.0 | 7.3 | 6.2 | 9.9 |
| RP11-367G18.1 | 0.5 | 0.5 | 0.5 | 0.4 | 0.8 | 1.1 | 0.9 | 1.3 |
| MARCKS | 13.6 | 12.8 | 13.6 | 14.1 | 14.5 | 16.4 | 12.9 | 19.0 |
| ULBP3 | 0.3 | 0.3 | 0.4 | 0.5 | 0.6 | 0.5 | 0.5 | 0.8 |
| KLF7 | 0.8 | 0.9 | 0.8 | 0.8 | 1.5 | 1.4 | 1.4 | 1.9 |
| CHPF | 7.6 | 7.0 | 5.2 | 7.3 | 14.6 | 15.5 | 12.6 | 16.0 |
| TMX1 | 3.9 | 3.3 | 3.6 | 3.8 | 3.8 | 3.6 | 3.8 | 4.7 |
| OSBPL8 | 1.3 | 1.3 | 1.3 | 1.3 | 1.4 | 1.3 | 1.3 | 1.5 |
| PPIF | 9.1 | 9.8 | 8.1 | 7.8 | 27.0 | 30.9 | 19.0 | 22.0 |
| SF3A3 | 5.2 | 4.6 | 5.1 | 5.5 | 5.3 | 5.1 | 5.1 | 5.5 |
| DHTKD1 | 2.2 | 2.5 | 2.3 | 1.9 | 1.4 | 1.4 | 1.6 | 1.7 |
| RNU4-2 | 0.4 | 0.5 | 2.8 | 0.3 | 0.6 | 0.4 | 1.2 | 1.2 |
| EPT1 | 1.5 | 1.6 | 1.5 | 1.5 | 1.7 | 1.8 | 1.8 | 1.9 |
| ZYG11B | 0.9 | 1.0 | 1.0 | 0.9 | 0.8 | 0.8 | 0.8 | 0.8 |
| THAP1 | 0.9 | 0.9 | 0.9 | 0.9 | 0.9 | 0.9 | 0.9 | 0.8 |
| RCAN3 | 1.2 | 1.1 | 1.2 | 1.5 | 1.2 | 1.2 | 1.2 | 1.2 |
| ETV1 | 0.2 | 0.2 | 0.2 | 0.5 | 0.2 | 0.1 | 0.2 | 0.2 |
| CD74 | 231.6 | 211.0 | 229.2 | 136.1 | 154.1 | 124.5 | 112.1 | 97.9 |
| ISOC2 | 4.2 | 3.8 | 3.3 | 4.2 | 4.2 | 3.6 | 4.4 | 3.8 |
| CEP131 | 2.2 | 1.8 | 2.2 | 2.5 | 1.8 | 1.5 | 1.9 | 1.6 |
| ERI1 | 1.0 | 0.8 | 0.9 | 0.8 | 0.7 | 0.7 | 0.7 | 0.6 |
| DERL3 | 2.0 | 1.4 | 1.6 | 1.7 | 1.6 | 1.6 | 1.8 | 1.4 |
| CKMT1B | 1.4 | 1.1 | 1.1 | 1.4 | 1.3 | 1.4 | 1.5 | 1.2 |
| FOXJ1 | 4.6 | 3.2 | 7.2 | 0.8 | 0.5 | 0.2 | 0.3 | 0.2 |
| TNFRSF11B | 0.2 | 0.2 | 0.2 | 2.2 | 0.2 | 0.4 | 0.1 | 0.1 |
| HLA-DPB1 | 21.6 | 25.3 | 24.3 | 15.6 | 21.3 | 17.6 | 18.3 | 11.2 |
| SAMD10 | 1.2 | 1.5 | 1.4 | 1.2 | 0.8 | 0.9 | 0.8 | 0.5 |
| CD79A | 1.3 | 0.6 | 1.1 | 1.1 | 2.6 | 1.5 | 2.6 | 1.1 |
| IGKV1-39 | 0.4 | 0.1 | 0.2 | 0.1 | 0.7 | 0.5 | 0.9 | 0.3 |
| MSMB | 2.4 | 1.6 | 2.0 | 1.2 | 5.5 | 3.5 | 5.5 | 0.4 |

**^*^** The average FPKM value (×10^5^) of genes in different CESC and HNSC patient groups with or without complete response to radiotherapy. PIK3CA-wt, wild type PIK3CA; PIK3CA-alt, mutated or amplificated PIK3CA. “CR” or “no CR”, complete response or not complete response.
